# Meals of differing caloric content do not alter physical activity behavior during a subsequent simulated recess period in children

**DOI:** 10.1186/s40064-016-2160-2

**Published:** 2016-04-22

**Authors:** Kelly J. Smith, Rachael Pohle-Krauza, Samantha Uhas, Jacob E. Barkley

**Affiliations:** Kent State University, 163E MACC Annex, 350 Midway Drive, Kent, OH 44242 USA; Youngstown State University, Youngstown, OH USA

**Keywords:** Childhood obesity, School lunch, Children’s physical activity, Caloric intake

## Abstract

**Objective:**

Research on adults and animals has demonstrated that chronic and acute overfeeding can alter physical activity behavior. However, there are no assessments of the acute effects of high-calorie (HC) meals on physical activity behavior in children. This is of importance as a typical school lunch is HC. If this type of meal negatively impacts subsequent physical activity behavior, the ability of post-lunch recess periods as a means to increase energy expenditure may be lessened.

**Purpose:**

To assess the effect of two meals of differing caloric content, HC and low calorie (LC), on children’s subsequent physical activity behavior.

**Methods:**

Nineteen healthy children (aged 6–10) completed two laboratory sessions where they were fed lunch with HC or LC content, but equivalent macronutrient distribution. Children had 15 min to consume as much of the meal as possible per session. Children consumed 659.5 ± 101.3 kcal in the HC condition and 291.8 ± 12.1 kcal in the LC condition. After the meal, children went to a gymnasium for 40 min. In the gymnasium children had free-choice access to obstacle courses, various sports equipment, and a table with sedentary activities. Children could play with any of the activities in any amount they wished for the entire activity session. Children’s physical activity was monitored with accelerometers and that data was converted into caloric expenditure. Each child ate all meals and participated in the free-choice activity sessions with no other children present.

**Results:**

Caloric expenditure during the free-choice activity sessions was not significantly different (*p* = 0.4) between the HC (89.2 ± 27.3 kcals) and LC (83.4 ± 34.9 kcals) conditions. However, caloric balance (kcals eaten–kcals expended) was 2.74-fold greater (*p* < 0.001) in the HC condition (Δ 570.3 ± 92.2 kcals) than the LC condition (Δ 208.4 ± 32.0 kcals).

**Conclusion:**

Children did not alter their physical activity behavior during a free-choice activity session after consuming a HC meal versus a LC meal. Because activity was not different across the two conditions, children had a much greater caloric surplus during the HC condition than the LC condition.

## Background

Childhood obesity has become an epidemic in many countries worldwide and current data from the Centers for Disease Control indicate that more than one third of American children are overweight or obese (Clark et al. 2015; Ogden et al. 2014). Regular participation in physical activity is inversely associated with the development of childhood obesity (Ball et al. 2005; Collings et al. 2013). Unfortunately, there is evidence that the majority of American children do not participate in the recommended amounts of moderate or vigorous intensity physical activity (CDC 2014; Pate et al. 2002). In addition to leading to the development of obesity in childhood a lack of participation in adequate physical activity has been associated with negative health consequences. Children who are overweight or obese are more prone to impaired glucose tolerance and insulin resistance which are known precursors to the development of type 2 diabetes (Mohsin et al. 2012; Whitlock et al. 2002). Aside from health consequences, obese children also have a greater risk of exhibiting social and psychological problems due to a poor self-esteem, low self-worth and negative peer interactions (Small and Aplasca 2016; Janssen et al. 2004; Schwartz and Puhl 2003). Because of the seriousness of the consequences of a physical inactivity in youth there is a need for a better understanding of the factors influencing physical activity behavior in children. This greater understanding could aid in the development of interventions and recommendations which maximize physical activity participation in youth.

In addition to a lack of adequate physical activity, children today consume historically excessive amounts of calories and dietary fat which is also a major contributor to the development of obesity (Drewnowski and Specter 2004; Pereira et al. 2013). Food that is high in calories and fat is more readily available than it has been in past years which may contribute to a larger caloric intake (Popkin and Hawkes 2016; Raynor et al. 2004). In addition, there is evidence that this excessive caloric consumption may be associated with children’s leisure activities (Epstein et al. 2005). For example, participation in sedentary behavior is positively associated with energy intake in children and interventions designed to reduce sedentary behaviors also lead to a reduction in caloric intake (Epstein et al. 2005). While this may support the notion that sedentary behavior affects diet, there is little research that evaluates the effects of diet on physical activity behavior in children.

Physiologically, it is possible that a large meal could impact physical activity as exercising muscle will compete with the gut for oxygenated blood flow after a meal (Eriksen and Waaler 1994). Basic animal studies have demonstrated that feeding rats a high-fat/high-calorie chow reliably decreases physical activity behavior relative to those that are fed standard chow (Bjursell et al. 2008). However, the research examining the effects of diet on physical activity behavior in humans is limited to adults and is equivocal (Levine et al. 1999). Levine et al. (1999) demonstrated that adults who were over-fed for a period of 8 weeks had one of two responses as it pertained to physical activity, increased physical activity [primarily non-exercise activity thermogenesis (NEAT)] and no weight gain or decreased (or maintained) physical activity and weight gain. This indicated that some individuals respond to overfeeding like the laboratory animal model by decreasing activity and gaining weight while other had the opposite response. In addition to having equivocal findings and examining adults only, this previous research did not assess the acute effects of a single high-calorie meal on subsequent physical activity.

Understanding the acute effects of over-feeding on subsequent physical activity is particularly relevant to school-aged children as this is a scenario many children encounter on a daily basis at school. School lunches are typically high in fat and energy rich (Addison et al. 2006) and while there are equivocal findings of the benefits (e.g., fewer behavioral problems, increased subsequent fruit, vegetable, calcium consumption) of a physically-active recess period preceding school lunches (Bergman et al. 2004; Fenton et al. 2015; Getlinger et al. 1996; Tanaka et al. 2005) such recess periods usually occur after students finish eating (Jago and Baranowski 2004). During this typical post-lunch recess period children are often encouraged to be physically active either in a gymnasium or outside on a school playground or ball field (Jago and Baranowski 2004). This period of recess is an excellent opportunity for children to be physically active, however the high-calorie/high-fat school lunch may impair children’s subsequent ability to be physically active and their enjoyment of that activity. Because physical education classes are increasingly being removed from schools, post-lunch recess periods may be the only opportunity for in-school physical activity for many children (Verstraete et al. 2006). Therefore, understanding factors that may affect physical activity during these recess periods is of importance. However, while the evidence from animal research, the physiologic responses known to occur post-meal and, to a lesser extent, the research by Levine et al. (1999) on adults provide evidence that diet may affect physical activity behavior there are no studies assessing the acute effects of eating meals of differing caloric content on subsequent physical activity behavior in children.

### Purpose

The purpose of this study was to test the effect of eating two meals differing in caloric content, one high calorie (HC) and one low calorie (LC), on subsequent voluntary physical and sedentary activity in children during a simulated recess period. We hypothesized that consuming the HC meal would reduce subsequent physical activity behavior and liking (i.e., enjoyment) of that activity relative to consuming the LC meal in children.

## Methods

### Participants

Children from the ages of 6–10 years who were free of any contradictions to physical activity (e.g., orthopedic, metabolic, cardiovascular disorders) were eligible to participate in the study. Eligibility was determined via phone interview with each child’s parent or guardian. The parents or guardians were asked to review a list of foods to be used in the study and indicate any dislikes or allergies their child may have. If the child indicated an unwillingness to consume any of the test foods (due to allergy or dislike) they were not able to participate. A total of 19 participants (*n* = six girls, one Hispanic, two African Americans, 16 Caucasians) were recruited via flyers posted in the local community and from a database of participants who had previously contacted the laboratory for separate, unrelated studies. After the child was cleared to participate they reported to the laboratory on two separate occasions. During the first visit, participants and their parent/guardians completed assent and consent forms, respectively. Subsequently, anthropometric measurements of height and weight were performed using a stadiometer (Charder Medical, Da Li City, Taiwan, China) and balance beam scale (Health O Meter, Alsip, IL). Body mass index (BMI, kg m^−2^) was then calculated. The participant then completed the first of two experimental conditions. This study was approved by the University Institutional Review Board.

### Study design

The study utilized a within-subjects’ design with all participants completing two, separate, midday, 2-h experimental sessions during which children ate a meal and then participated in a physical activity session. There was a washout period of at least 24 h between sessions. The energy content of the meal (i.e., lunch) during each session was manipulated (HC, LC). While all participants completed both conditions, each child was randomly assigned to one of the two possible treatment orders (HC then LC or LC then HC) using a random numbers generator (i.e., treatment order was randomized). After the meal, children had free access to physical and sedentary activities for a period of 40 min, during which physical activity and sedentary behavior was monitored by an accelerometer and stopwatch, respectively. Because the presence of other children and adult guardians may affect physical activity behavior in youth, all participants underwent test session procedures unaccompanied by anyone except research staff (Sanders et al. 2014; Rebold et al. 2014).

### Laboratory visit: feeding portion

During each of the two study visits participants reported to the laboratory at the same time (11 am or 12 pm) and were asked eat a similar breakfast with no other snacks consumed after breakfast/before lunch in order to reduce the effects of variations in pre-session meal composition on food intake on study visit days. The parents/guardians of all participants indicated that they ate similar breakfasts and abstained from snacks before coming to the lab.

### Test meals

When the participant reported to the laboratory they were presented with either their HC or LC meal. The two meals consisted of the same foods (Wendy’s^®^ chicken nuggets, Wendy’s^®^ French fries, Hawaiian^®^ punch, and ketchup) in different amounts. Current dietary guidelines specify that daily energy requirements for children (aged 6–10) range from 1600 to 2200 kcal day^−1^ (United States Department of Health 2010), consistent with studies employing double labeled water methods, showing calorie needs for this age group to range from 1600 to 2500 kcal day^−1^ (Torun 2005). Thus, our test meals (730 or 315 kcal), provided approximately 35 or 15 % of estimated daily needs (assuming 1900 kcal day^−1^ diet). Each experimental meal was composed of the same dietary percentages, 9 % of daily protein, 47 % of daily carbohydrates, and 44 % of recommended daily fat intake. The energy provided was less than (LC meal) or equal to (HC meal) those provided in an average school lunch for children in this age group (Addison et al. 2006). All foods were weighed, using a digital scale, before serving. The HC meal contained 730 kcals and the LC meal contained 315 kcal or 40 % of the calories from the HC condition. Table 1 describes the composition of the two meal conditions.

**Table 1:** Composition of test meals

	Purchase portion	Weight (g)	Energy (kcal)	Protein (g)	Carbohydrate (g)	Fat (g)
*Higher calorie*						
Wendy’s chicken nuggets	(6 pieces)	90	264	12	15.6	16.8
Wendy’s French fries	(~a large)	110	325.4	3.9	42.6	16.3
Hawaiian punch	(10 oz)	_	87.5	0	21.3	0
Total			676.9	15.9	79.5	33.1
% Energy from macronutrients				9	47	44
*Lower calorie*						
Wendy’s chicken nuggets	(3 pieces)	36	105.6	4.8	6.2	6.7
Wendy’s French fries	(~a large)	44	130.2	1.6	17.1	6.5
Hawaiian punch	(4 oz)	_	35	0	8.5	0
Total			270.8	6.4	31.8	13.2
% Energy from macronutrients				9	47	44
Ketchup	(1 tub)	27	30	0	8	0

The participants were encouraged to consume 100 % of the foods and beverages presented at the meal, and asked to complete consumption within 20 min. Research personnel encouraged the children to consume their entire meal using the following language: “It is important that you eat as much of this food as possible. We want you to try your best to eat all of the food in front of you.” If a child was unwilling/unable to eat their entire experimental meal, the remaining food was weighed using a digital scale to determine the total caloric intake for that session. While the child consumed their meal they were allowed to watch an age-appropriate video of their choice (SpongeBob Squarepants^®^ or Scooby Doo^®^). The age-appropriate video was chosen to prevent participant boredom and increase the likelihood of the child consuming their entire meal (Temple et al. 2007).

### Laboratory visit: gymnasium portion

Immediately after children completed their meal, they were taken into a 4300 square foot gymnasium located in the same building as the laboratory. In the gymnasium children had free access to physical and sedentary activities for a period of 40 min. The child was alone in the gymnasium for the entire 40 min except for the laboratory personnel. Physical activity equipment included; five, one foot (0.305 m) tall modified hurdles, jump rope, several Nerf™ footballs and flying discs with targets and goals (Hasbro, Pawtucket, Rhode Island), standing long jump, kicking a soccer ball around a series of seven cones, shooting a basketball at a standard 10 feet (3.05 m) hoop and navigating an obstacle course made up of gymnastic/soft-play equipment (UCS inc. Lincolnton, NC). A table and chair was also placed in the gymnasium for the children to participate in sedentary activities. Those activities included drawing, word finds, books, and table top games. If the child chose to participate in sedentary activities they had to be seated in the chair. The child was able to do any activities, in any pattern, for the entire 40 min session.

### Measures

#### Physical activity

During the 40 min gymnasium sessions the participant wore a validated accelerometer (Actigraph, GT1M) (Freedson et al. 2005). Total per-minute accelerometer counts were converted to metabolic equivalents (METs, 1 MET = 3.5 ml kg^−1^ min^−1^ VO_2_) and energy (i.e., caloric) expenditure using validated methods (Freedson et al. 2005). Accelerometer counts, METs, and caloric expenditure where the measures of physical activity.

### Sedentary behavior

The amount of time children allocated to the table of sedentary activities was recorded via a stopwatch (Traceable^®^ Stopwatch, Fisher Scientific, Waltham, Massachusetts). A bout of sedentary time was initiated when a child elected to sit at the chair where the sedentary activities were located and ended when the child left that chair to return to the physical activities. The time for all bouts (if there was more than one) of sedentary activity during the 40 min gymnasium period was summed. This total time (min) allocated to sedentary activities was the measure of sedentary behavior and is similar to methods utilized previously (Barkley et al. 2014; Sanders et al. 2014).

### Perceived exertion

At the conclusion of each 40-min activity session children reported their ratings of perceived exertion (RPE). RPE was assessed via the validated, pediatric OMNI walk/run scale which uses numeric and verbal descriptors of fatigue ranging from 0 “not tired at all” to 10 “very, very tired” (Utter et al. 2002).

### Liking

The liking (i.e., enjoyment) of each physical activity session was assessed using a visual analog scale (VAS) designed to assessing the enjoyment of participating in an activity. This VAS consisted of a 100 mm line anchored by ‘do not like it at all’ on the left side and ‘like it very much’ on the right side. Children were asked to mark a point on the line that corresponds to their liking of that activity session. Liking, or hedonics, is an affective rating of a behavior that is positively associated with physical activity participation in youth (Roemmich et al. 2008).

### Analytic plan

Participant physical characteristics (age, height, weight, BMI) in boys and girls were compared using independent samples t tests. Because there were differences in these physical characteristics and there are established sex difference in physical activity behavior in youth, sex was included as an independent variable in the subsequent analyses (Van Der Horst et al. 2007). Two sex (boys, girls) by two condition (LC, HC) analyses of variance (ANOVAs) with repeated measures on condition were used to assess differences in caloric intake and energy expenditure, liking and RPE during the activity sessions. An additional sex by condition ANOVA was also performed assessing the difference of the caloric balance (caloric balance = kcals consumed during lunch − kcals expended during simulated recess). Post hoc comparisons for any significant interactions were performed via t tests. All data were analyzed using SPSS version 18 (Evenston, IL). NOTE: caloric expenditure was calculated from the accelerometer counts. Therefore, we did not include accelerometer counts in the final analysis as this would yield the same result as any variable calculated from it.

## Results

### Physical characteristics

There was significant (*p* = 0.004) difference of ages between boys (8.6 ± 1.1 years old) and girls (6.7 ± 1.1 years old). There was also a significant (*p* = 0.026) difference in height between the boys (136.3 ± 7.7 cm) and girls (125.2 ± 12.0 cm). There were no significant differences between boys and girls for weight (*p* ≥ 0.16) (boys, 32.6 ± 9.6 kg, (girls, 26.1 ± 6.5 kg) or BMI (boys, 17.2 ± 3.1 kg m^−2^, girls, 16.4 ± 1.0 kg m^−2^).

### Caloric intake

As expected, there was a significant (*p* < 0.001) main effect of condition for calories consumed. Children consumed more calories in the HC condition (659.5 ± 101.3 kcal) than the LC condition (291.8 ± 12.1 kcal). There were no main or interaction effects (*p* ≥ 0.25) of sex.

### Caloric expenditure

There were no main or interaction effects (*p* ≥ 0.17) of condition for caloric expenditure. Participant’s expended similar amounts of kcals across the two conditions (83.4 ± 35.0 kcals or 4.4 METs LC, 89.2 ± 27.3 kcals or 4.6 METs HC). There was a trend (*p* = 0.057) towards main effect of sex as, overall, boys (94.8 ± 31.9 kcals or 4.7 METs) expended a greater number of kcals than girls (68.1 ± 20.85 kcals or 4.2 METs).

### Caloric balance

**Fig. 1:**
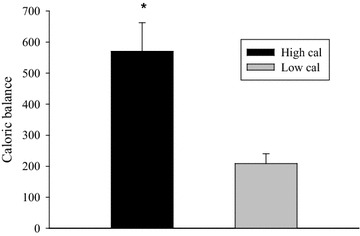
Illustrates a significantly greater caloric surplus (kcals consumed–kcals expended) in the high calorie condition versus the low calorie condition. *p < 0.001

There was a significant (*p* < 0.001) main effect of condition for caloric balance. Caloric balance was 2.74-fold greater in the HC condition (Δ 570.3 ± 92.2 kcals) than the LC condition (Δ 208.4 ± 32.0 kcals) (Fig. 1). There were no main or interaction effects (*p* ≥ 0.19) of sex.

### Liking

There was a significant (*p* = 0.044) sex by condition interaction for liking. The girls had a greater liking for the activity session after the HC meal (94.7 ± 4.9 mm) versus the LC meal (79.2 ± 21.6 mm) whereas the boys did not alter liking across conditions (84.2 ± 17.2 mm HC, 85.9 ± 13.2 mm LC). There were no main effects (*p* ≥ 0.10) of condition or sex.

### RPE

There was a trend (*p* = 0.058) towards a main effect of condition for RPE. Children reported a greater RPE in the physical activity period after the LC meal (4.4 ± 2.5) than after the HC meal (3.6 ± 2.5). There were no main or interaction effects (*p* ≥ 0.27) of sex.

## Discussion

The purpose of this study was to assess the effect of meals of differing caloric content, HC and LC, on children’s subsequent physical activity behavior during a simulated recess period. While there is evidence that physical activity preceding school lunch leads to increased intake of certain nutrients (e.g., Iron, Calcium vitamins A and C) and may reduce both behavioral problems and the amount of food that is wasted (Bergman et al. 2004; Fenton et al. 2015; Getlinger et al. 1996; Tanaka et al. 2005), this was the first assessment of the acute impact of meals of differing calorie content on subsequent physical activity in children. Understanding the acute effects of these meals on physical activity could provide additional support for more healthful meals in school cafeterias in an effort not just to reduce caloric intake but also maximize physical activity participation during recess periods in children. While previous research has reported equivocal findings on the effect of overfeeding on physical activity in adults (i.e., some decreased physical activity, others increased) we hypothesized that a HC meal would decrease children’s physical activity relative to a LC meal (Levine et al. 1999). Contrary to this hypothesis, our results indicated that meals of differing caloric content did not alter physical activity behavior during a subsequent simulated recess period in children.

Our hypothesis was based largely upon previous basic animal studies which showed feeding rats a high-fat/high-calorie chow decreased physical activity behavior relative to rats that are fed standard chow (Bjursell et al. 2008). However, children’s physical activity level remained the same during both conditions. While this would suggest that there was no acute effect of differential caloric consumption on physical activity in children, the present results yield a potentially troubling finding. Children had a surplus (kcals consumed–kcals expended) of 362 kcals more in the HC condition than the LC condition. This would suggest that children did not alter their physical activity in the HC condition to compensate for the additional caloric intake. Providing children with a recess period after a school lunch is likely not adequate to justify the excessive caloric content of a school meal. Children do not appear to be willing or capable to “burn off” these excess calories during recess and this could have a potent obesogenic effect.

The effects of the meal condition on the liking and RPE for the activity sessions were also unexpected. Presently, girls had a greater liking for the physical activity session following the HC meal versus the LC meal whereas boys did not alter their liking across conditions. There was also a trend towards a main effect of condition for RPE. Children reported a greater RPE for the physical activity period after the LC meal than after the HC meal. While the greater RPE in the LC condition may explain why girls had a greater liking for the HC condition (i.e., they perceived it as less difficult), it is not clear why children would perceive the HC condition activity as less difficult than the LC condition. Because the work of digestion would be greater after the HC meal (i.e., greater amounts of blood to the gut) we had hypothesized that RPE would be greater in this condition than the LC condition (Bjursell et al. 2008; Levine et al. 1999). Perhaps the caloric content of the LC meal, relative to the HC meal, was inadequate, leaving children with a greater appetite and therefore, less comfortable during their subsequent physical activity session. While these results are puzzling, the differences in liking and RPE did not manifest into differences in physical activity behavior across the conditions.

While there are intriguing results reported herein, there are also limitations present. Our sample size was small (*N* = 19) and was composed of healthy children. Levine noted that overweight and lean adults may have differential responses to overfeeding, perhaps the same is true of children (Levine et al. 1999). Examining a larger sample and comparing groups of overweight/obese and non-overweight children would allow us to examine if overweight/obese children respond differently across meal conditions. Additionally, children were only fed one meal on two different occasions. Examining the effects of HC and LC meals over a longer period of time (e.g., several days) may yield different effects than what is noted presently. It would also be beneficial for future studies to monitor physical activity and food consumption throughout the entire day rather than just during a 40 min period. Monitoring children’s physical activity throughout the day would also allow us to determine if the caloric content of the HC versus LC meals had a delayed effect on physical activity that occurs later in the day. This may be important as previous animal studies noted significant declines in physical activity beginning hours after the initiation of a high-fat and high-calorie diet (Bjursell et al. 2008).

## Conclusion

In conclusion, contrary to our hypothesis children were not more physically active during a free-choice activity session in a gymnasium after consuming a LC meal versus a HC meal. However, because activity was not different after either meal condition, children had a much greater caloric surplus during the HC condition than the LC condition. Children did not compensate for the extra calories consumed during the HC meal with additional subsequent physical activity. This large caloric surplus in the HC condition may have important obesogenic implications in that when children are fed a typical school lunch, which is HC, they may have a very large caloric surplus even after participating a recess period. Future research examining ways to maximize physical activity during in-school recess is warranted. One possible option would be to explore the effect or pre-lunch versus post-lunch recess on physical activity behavior during said recess period. Previous studies have reported that pre-lunch recess, relative to post-lunch recess, increases subsequent healthful nutrient consumption during lunch. However, the effect of pre-lunch recess upon physical activity behavior is heretofore unknown.

## References

[CR1] Addison CC, Jenkins BW, White MS, Young L (2006). Examination of the food and nutrient content of school lunch menus of two school districts in Mississippi. Int J Environ Res Public Health.

[CR2] Ball GD, Marshall JD, McCargar LJ (2005). Physical activity, aerobic fitness, self-perception, and dietary intake in at risk of overweight and normal weight children. Can J Diet Pract Res.

[CR3] Barkley JE, Salvy SJ, Sanders GJ, Dey S, Von Carlowitz KP, Williamson ML (2014). Peer influence and physical activity behavior in young children: an experimental study. J Phys Act Health.

[CR4] Bergman EA, Buergel NS, Englund T, Femrite A (2004). The relationship of meal and recess schedules to plate waste in elementary schools. J Child Nutr Manag.

[CR5] Bjursell M, Gerdin AK, Lelliott CJ, Egecioglu E, Elmgren A, Tornell J, Oscarsson J, Bohlooly Y (2008). Acutely reduced locomotor activity is a major contributor to Western diet-induced obesity in mice. Am J Physiol Endocrinol Metab.

[CR6] Centers for Disease Control (2014) Youth Risk Behavior Surveillance—United States, 2013. *MMWR* 63(SS-4) 24918634

[CR7] Clark BR, White ML, Royer NK, Burlis TL, DuPont NC, Wallendorf M, Racette SB (2015). Obesity and aerobic fitness among urban public school students in elementary, middle, and high school. PLoS ONE.

[CR8] Collings PJ, Brage S, Ridgway CL, Harvey NC, Godfrey KM, Inskip HM, Cooper C, Wareham NJ, Ekelund U (2013). Physical activity intensity, sedentary time, and body composition in preschoolers. Am J Clin Nutr.

[CR9] Drewnowski A, Specter SE (2004). Poverty and obesity: the role of energy density and energy costs. Am J Clin Nutr.

[CR10] Epstein LH, Roemmich JN, Paluch RA, Raynor HA (2005). Influence of changes in sedentary behavior on energy and macronutrient intake in youth. Am J Clin Nutr.

[CR11] Eriksen M, Waaler BA (1994). Priority of blood flow to splanchnic organs in humans during pre- and post-meal exercise. Acta Physiol Scand.

[CR12] Fenton K, Rosen NJ, Wakimoto P, Patterson T, Goldstein LH, Ritchie LD (2015). Eat lunch first or play first? Inconsistent associations with fruit and vegetable consumption in elementary school. J Acad Nutr Diet.

[CR13] Freedson P, Pober D, Janz KF (2005). Calibration of accelerometer output for children. Med Sci Sports Exerc.

[CR14] Getlinger M, Laughlin C, Bell E, Akre C, Arjmandi B (1996). Food waste is reduced when elementary school children have recess before lunch. J Am Diet Assoc.

[CR15] Jago R, Baranowski T (2004). Non-curricular approaches for increasing physical activity in youth: a review. Prev Med.

[CR16] Janssen I, Craig WM, Boyce WF, Pickett W (2004). Associations between overweight and obesity with bullying behaviors in school-aged children. Pediatrics.

[CR17] Levine JA, Eberhardt NL, Jensen MD (1999). Role of nonexercise activity thermogenesis in resistance to fat gain in humans. Science.

[CR18] Mohsin F, Mahbuba S, Begum T, Azad K, Nahar N (2012). Prevalence of impaired glucose tolerance among children and adolescents with obesity. Mymensingh Med J.

[CR19] Ogden CL, Carroll MD, Kit BK, Flegal KM (2014). Prevalence of childhood and adult obesity in the United States, 2011–2012. J Am Med Assoc.

[CR20] Pate RR, Freedson PS, Sallis JF, Taylor WC, Sirard J, Stewart TG, Dowda M (2002). Compliance with physical activity guidelines: prevalence in a population of children and youth. Ann Epidemiol.

[CR21] Pereira HR, Bobbio TG, Antonio MÂ, Barros Filho Ade A (2013). Childhood and adolescent obesity: how many extra calories are responsible for excess of weight?. Rev Paul Pediatr.

[CR22] Popkin BM, Hawkes C (2016). Sweetening of the global diet, particularly beverages: patterns, trends, and policy responses. Lancet Diabetes Endocrinol.

[CR23] Raynor HA, Polley BA, Wing RR, Jeffery RW (2004). Is dietary fat intake related to liking or household availability of high- and low-fat foods?. Obes Res.

[CR24] Rebold MJ, Lepp A, McDaniel J, Kobak MS, Barkley JE (2014) The experimental effect of parental influence on children’s physical activity. In: Medicine and science in sport and exercise (5S) supplement. 62nd meeting in American College of Sports. Medicine, San Diego, CA

[CR25] Roemmich JN, Barkley JE, Lobarinas CL, Foster JH, White TM, Epstein LH (2008). Association of liking and reinforcing value with children’s physical activity. Physiol Behav.

[CR26] Sanders GJ, Peacock CA, Williamson ML, Wilson K, Carnes A, Barkley JE (2014). The effect of friendship groups on children’s physical activity: an experimental study. J Behav Health.

[CR90] Schwartz MB, Puhl R (2003). Childhood obesity: a societal problem to solve. Obes Rev.

[CR27] Small L, Aplasca A (2016). Child obesity and mental health: a complex interaction. Child Adolesc Psychiatr Clin N Am.

[CR28] Tanaka C, Richards KL, Takeuchi LSL, Otani M, Maddock J (2005). Modifying the recess before lunch program: a pilot study in Kaneohe elementary school. Calif J Health Promot.

[CR92] Temple JL, Giacomell AM, Kent KM, Roemmich JN, Epstein LH (2007). Television watching increases motivated responding for food and energy intake in children. Am J Clin Nutr.

[CR29] Torun B (2005). Energy requirements of children and adolescents. Public Health Nutr.

[CR30] United States Department of Health and Human Services (2010) US Department of Agriculture. Dietary Guidelines for Americans

[CR31] Utter AC, Robertson RJ, Nieman DC, Kang J (2002). Children’s OMNI Scale of Perceived Exertion: walking/running evaluation. Med Sci Sports Exerc.

[CR32] Van Der Horst K, Paw MJ, Twisk JW, Van Mechelen W (2007). A brief review on correlates of physical activity and sedentariness in youth. Med Sci Sports Exerc.

[CR91] Verstraete SJ, Cardon GM, De Clercq DL, De Bourdeaudhuij IM (2006). Increasing children's physical activity levels during recess periods in elementary schools: the effects of providing game equipment. Eur J Public Health.

[CR33] Whitlock EP, Orleans CT, Pender N, Allan J (2002). Evaluating primary care behavioral counseling interventions: an evidence-based approach. Am J Prev Med.

